# Simulating Interventions for Symptoms Linking Problematic Social Networking Sites Use to Online Aggressive Behavior Among Chinese College Students: A Gender‐Differentiated Network Analysis

**DOI:** 10.1002/pchj.70085

**Published:** 2026-03-01

**Authors:** Haiqun Niu, Xiaoxu Lu, Yichao Lv, Jie Gui, Shujian Wang, Yanqiang Tao, Jingyi Zhou

**Affiliations:** ^1^ School of Education and Psychology, Shaoxing University Shaoxing China; ^2^ Collaborative Innovation Center of Assessment for Basic Education Quality, Beijing Normal University Beijing China; ^3^ Faculty of Psychology, Beijing Normal University Beijing China; ^4^ Beijing Key Laboratory of Applied Experimental Psychology, National Demonstration Center for Experimental Psychology Education Beijing China; ^5^ Faculty of Architectural Decoration and Art, Jiangsu Vocational College of Electronics and Information Huaian China; ^6^ Shanghai Key Laboratory of Mental Health and Psychological Crisis Intervention (ECNU), School of Psychology and Cognitive Science, East China Normal University Shanghai China; ^7^ College of Humanities and Development, China Agricultural University Beijing China

**Keywords:** college students, online aggressive behavior, problematic social networking sites use, symptom network

## Abstract

Prior empirical evidence supports the close association between problematic social networking sites use (PSNS) and online aggressive behavior (OAB). However, few studies have examined the potential symptom connection between these two issues as part of a multidimensional mechanism. The current study aims to explore the underlying network structure between PSNS use and OAB and conduct a simulated intervention using the NodeIdentifyR algorithm (NIRA), taking gender into account to further inform the implementation of intervention measures. A total of 1325 participants completed questionnaires that assessed SNS addictive tendencies and online aggression. The symptom‐specific intervention simulation analysis was conducted to clarify which symptoms can alleviate or exacerbate the overall performance of PSNS and OAB. Findings indicated that there were significantly different symptom connections in both groups, with no symptom linked to the two behaviors in the male group, while “insomnia” and “instrumental overt aggression” linked the two in the female group (*p* < 0.05). In addition, “dual existence” may be the most effective alleviating intervention target for both groups (NIRA = 1.34 for males and NIRA = 1.44 for females). Besides, “virtual friend anxiety” and “online relationship satisfaction” should be considered separately for preventive care when dealing with males (NIRA = 1.07) and females (NIRA = 1.37). The findings offer significant implications for gender‐specific strategies to alleviate PSNS use and OAB linkage.

## 1 Introduction

The problematic social networking sites use (PSNS) is often manifested as social networking site (SNS) users having difficulty controlling their usage patterns, frequently checking their accounts, and constantly wondering when they can use SNS again (Andreassen 2015). Although the prevalence of PSNS varies among populations and studies, the results from a meta‐analysis of 63 independent samples of approximately 35,000 participants from 32 countries suggest that the average prevalence is estimated to be 5% (Cheng et al. 2021). Various physical and mental impairments, including depression, family conflict, and substance use (Andreassen 2015; Liu et al. 2011; Milošević‐Đorđević and Žeželj 2014), have been proven to be linked to PSNS. Without parental supervision, college students' use of SNSs and online misbehaviors may be more serious (Manago et al. 2012; Nocera and Dahlen 2020). Therefore, there is a great need to find critical factors and interventions to reduce the problematic use of SNSs.

Recently, online aggressive behavior (OAB), typically expressed by intentionally posting offensive and threatening online content, or sharing maliciously false information on social media platforms to a person or group (Grigg 2010), has been found to be a closely related variable (Capurso et al. 2017; Darazi et al. 2023). It is widely accepted that SNS can enable and encourage OAB, as offenders can effortlessly reach their victims and bypass the restrictions that typically manage bullying in traditional environments (Chan et al. 2019). For instance, the study conducted by Erdur‐Baker (2010) reported that those who spent more time connected to SNS were more frequently involved in cyber‐aggressive behaviors. The deindividuation theory (Festinger et al. 1952) suggests that people tend to lose their inner restraints and have less self‐awareness and responsibility for their actions if they are anonymous. From this perspective, the anonymity and accessibility of SNSs diminish people's perceived sense of responsibility and psychological concerns about being caught, making them say things that they would not say face‐to‐face and easily behave in ways that hurt others by posting negative content or disseminating hostile rumors (Wen et al. 2023). Besides, from the perspective of social learning theory, people may also adopt aggressive behaviors if they have close associations with other online people who encourage cyberbullying behaviors (Piccoli et al. 2020). Those who are highly involved in SNSs receive a variety of information daily and are inevitably influenced by or identify with others' opinions and behaviors. In this way, they are prone to use aggressive language to attack another person directly (Rösner and Krämer 2016).

Moreover, empirical findings imply that different genders would exhibit different aggressive behaviors on SNSs. For instance, Dehue et al. (2008) found that boys were more likely to commit cyber overt aggression (i.e., hacking) through SNSs, whereas girls were more likely to use gossip (relational aggression). Other studies have also found that university girls have higher levels of relational aggression in cyber environments (Keith and Martin 2005; Nelson 2003). Wright (2020) further took masculine and feminine traits into account and indicated that boys and girls who endorsed more masculine traits engaged in more hacking on SNSs, while boys and girls with more feminine traits engaged in more cyber relational aggression. In addition, those who indulge in SNSs for different purposes also show different aggressive behaviors. For instance, García‐Fernández et al. (2022) concluded that high involvement in cyber‐gossiping was associated with online aggression behaviors, and adolescent girls were more involved in cyber‐gossiping than boys. Taken together, to gain a more nuanced understanding of the associations between PSNS and OAB, gender should be considered.

It is worth noting that most studies tend to link PSNS at the construct level with online aggression, as researchers often use standardized scales on PSNS, summing up responses to each item to create a total score, and then correlating that with other variables they want to measure (Hassan et al. 2023; Hou et al. 2023). Such a conventional approach helps produce basic correlations between different variables but will also blur the links between different symptoms (Beard et al. 2016). According to the network‐symptom theory of pathology (Borsboom 2017; Borsboom and Cramer 2013), mental disorders are caused by connections among symptoms (Cramer et al. 2010). Consistent with this framework, an expanding body of research has applied symptom network analysis to investigate problematic social media/internet use and its associations with mental health outcomes, demonstrating the value of disentangling symptom‐specific pathways (Cai et al. 2022; Luo et al. 2025; Peng and Liao 2023; Tao, Tang, Zou, et al. 2023). From this perspective, PSNS can be viewed as an entity consisting of symptoms like long online time, withdrawal (experiencing unpleasant feelings upon limiting or discontinuing SNS use), and mood modification (engagement in SNSs leads to a favorable change in emotional states) (Andreassen 2015). Thus, using the symptom network analysis (Borsboom and Cramer 2013), we can easily identify the main symptoms leading to the onset of the syndrome, as the links between PSNS and OAB can be seen clearly from nodes (representing symptoms) and edges (representing the relationship between the symptoms) presented within the network. For instance, insomnia may function as a bridge symptom in this relationship, as sleep disturbances may lead to prolonged smartphone use among young people during nighttime hours, and it simultaneously increases their susceptibility to aggressive behaviors (Bersani et al. 2022).

In a practical situation, however, the individual's pathological symptoms are subject to change and even recur after an intervention. Thus, it is more critical to see the actual effect of an intervention when targeting that specific symptom. Obviously, by analyzing the centrality indices of all nodes in the network, the symptom network analysis can only identify the key symptoms of disorders in a static way and does not allow for the visualization of treatment effects. Accordingly, the *NodeIdentifyR* algorithm (NIRA), the newly emerging network approach, was developed to simulate specific‐symptom clinical interventions for variables within a network (Lunansky et al. 2022). NIRA can generate projected networks by manipulating symptoms to trigger a series of reactions so that optimal prevention and intervention targets can be estimated based on the magnitude of network changes. Lunansky et al. (2022) applied NIRA to the PTSD assessment of participants who experienced the 2008 Wenchuan earthquake and modeled the effective treatment target of intrusive thinking, which was also confirmed to be a particularly important prevention target shortly thereafter.

Given the fact that results from existing research have not explained the underlying mechanism of symptom connection between PSNS and OAB well, let alone the use of simulation interventions to better predict which symptoms are the most critical from a dynamic perspective. The primary objective of this study is to bridge the gap by utilizing symptom network analysis and simulation intervention in the network model, while taking gender into account, to further elucidate the internal transmission mechanism between PSNS and OAB. By doing this, we try to determine (1) how the network structure linking PSNS and OAB differs between male and female groups and (2) which symptom has the greatest expected effect on reducing or increasing activation of the comorbidity of PSNS and OAB.

## 2 Materials and Methods

### 2.1 Participants and Procedure

The current study was conducted in Jiangsu province, China, from October to November 2023. The questionnaire was uploaded to the online questionnaire platform Wenjuanxing (https://www.wjx.cn), and a link to the questionnaire was sent to the students from several colleges in Huai'an City to collect data. All students provided electronically signed informed consent. Before collecting data, we estimated the required sample size in advance using a commonly accepted guideline in multivariate analysis—namely, having at least 10 participants per variable (Roscoe 1969). Since the total number of items across the scales was 37, we targeted a minimum sample size of 400 for each gender to ensure sufficient statistical power. A total of 1325 participants (age: *M* = 18.63, SD = 0.88) were actually recruited, and all were included in the final analysis, with no missing data. Of these, 745 were female (56.2%; age: *M* = 18.64, SD = 0.83) and 580 were male (43.8%; age: *M* = 18.62, SD = 0.94).

This study received review and approval from the ethical committee of Beijing Normal University (Reference number: 202305290090).

### 2.2 Measures

#### 2.2.1 Social Network Sites Addictive Tendencies Scale (SNSATS)

Social Network Sites Addictive Tendencies Scale (SNSATS), developed by Milošević‐Đorđević and Žeželj (2014), is an instrument used to assess social network sites' addictive behaviors. The SNSATS consists of six items that evaluate various manifestations of social network site addiction, including declining productivity, insomnia, dual existence, encroachment on other activities, online relationship satisfaction, and virtual friend anxiety. Responses are graded on a scale of 1 (“*Completely disagree*”) to 5 (“*Completely agree*”), with higher scores indicating more severe social network addiction. In the current study, the SNSATS exhibited good internal consistency, with a Cronbach's *α* value of 0.85.

#### 2.2.2 Adolescent Online Aggressive Behavior Scale (AOABS)

The Adolescent Online Aggressive Behavior Scale (AOABS) was developed by Li et al. (2011). The AOABS consists of two subscales, including instrumental aggression (15 items) and reactive aggression (16 items), and each subscale was divided into two factors: overt aggression and relational aggression. Participants should rate the frequency of their related behaviors on a 4‐point scale, where 1 indicates “*never*” and 4 indicates “*always*”. In this study, the Cronbach's *α* values of four dimensions in AOABS ranged from 0.89 to 0.95.

### 2.3 Analytic Plan

All statistical analyses were conducted using R software (version 4.3.2; R Core Team 2023). For six items of SNSATS and four dimensions of AOABS, we first conducted descriptive statistics on their continuous scores and independent sample *t*‐tests to examine gender differences using basic functions in R. Since the NIRA is designed specifically for Ising models (a network analysis for binary data), we binarized the SNSATS and AOABS scores based on their original continuous scores. Specifically, all variables were *Z*‐standardized, with scores recoded as 1 (indicating higher activation) when their *Z* value was ≥ 0, and as 0 (indicating lower activation) when their *Z* value was < 0. The dichotomized scores were also compared by gender.

#### 2.3.1 Ising Network Structure and Centrality Estimation

Before conducting network analysis, all items were evaluated for informativeness and redundancy following the recommendations of Marchetti (2019) and Mullarkey et al. (2019). The item check was conducted using the R package *networktools* (version 1.5.0; Jones 2022). The Ising network was modeled using the *estimateNetwork* function in the R package *bootnet* (version 1.6; Epskamp, Borsboom, and Fried 2018). This step applied logistic regression to estimate the parameters by regressing each binary variable on all other binary variables (van Borkulo et al. 2014). Here, edge weights and thresholds were two key parameters. Edge weights, deriving from the coefficients in the regression, represented the interrelations between nodes within the network. The threshold parameter, deriving from the intercept, represented the onset level of symptoms. A positive threshold value indicates that the symptom tends to become highly activated, whereas a negative threshold value reflects a tendency for the symptom to remain deactivated.

The Ising network was visualized using the *qgraph* (version 1.9.8; Epskamp et al. 2012). In this study, nodes in the network represented items from SNSATS or a dimension of AOABS, and the edges between nodes represented their relations. Green and red edges indicated positive and negative relations, respectively, with thicker edges denoting stronger relations (Borsboom and Cramer 2013). Then, using the *centralityPlot* function (Opsahl et al. 2010), we estimated the Expected Influence (EI) and bridge EI centrality indices for each node. EI was commonly used to measure a node's strength on the entire network structure. Bridge EI was used to determine whether a node plays a bridging role, linking two distinct mental communities (i.e., SNSATS and AOABS) within the network. Prior studies mainly focused on the node with the highest EI or bridge EI (Tao, Tang, Zou, et al. 2023).

In addition, we estimated separate male and female networks using the original continuous data to examine whether dichotomizing the variables altered the underlying relations among them. Specifically, we extracted the edge‐weight matrix from the continuous network and conducted a Mantel test (Fortin and Gurevitch 1993) to compare it with the matrix from the dichotomized network. If the high correlations observed indicated strong similarity, it suggests that dichotomization did not substantially affect the network structure.

#### 2.3.2 Network Stability and Accuracy

Using the R package *bootnet* (version 1.6; Epskamp, Waldorp, et al. 2018), we evaluated the accuracy and stability of the estimated network. Bootstrapping test on edges (*N* = 1000) was used to compute 95% confidence intervals (CIs) of edge weight, with narrow CIs indicating great accuracy of the edge estimates. The case‐dropping bootstrap test (*N* = 1000) was then conducted to calculate the correlation stability coefficient (CS‐C) to evaluate the stability of EI and bridge EI centralities. This coefficient indicates the maximum number of cases that can be removed while still retaining, with 95% probability, a correlation of at least 0.7 between statistics derived from the original network and those calculated from the reduced dataset. The CS‐C value was acceptable above 0.25 and preferably above 0.5 (Bringmann et al. 2019). Additionally, bootstrapped difference tests (*N* = 1000) were performed to investigate if the weight of two edges or the centrality of two nodes within the same network differs from one another (Epskamp, Borsboom, and Fried 2018).

#### 2.3.3 Network Comparison

Using the R package *NetworkComparisonTest* (NCT, version 2.2.2; van Borkulo et al. 2023), we explored the gender differences in four aspects of networks (iterations = 1000). NCT comprises four distinct tests: network structure invariance test (examines the differences in network structures), global strength invariance test (examines the differences in the total node strengths), edge strength invariance test (examines the differences in the local edge‐weight strengths), and centrality invariance test (evaluates differences in node centralities). Finally, we used Bonferroni correction to control for Type I error inflation due to multiple comparisons and reported the NCT results both with and without corrections. As a supplementary analysis, we also examined gender differences in network structure and global strength using continuous data networks.

#### 2.3.4 Simulation of Alleviating and Aggravating Interventions

Using the NIRA from the R package *IsingSampler* (version 0.2.3; Lunansky et al. 2022), we performed the simulation intervention analyses based on the above Ising networks. NIRA calculated sum scores to reflect the overall symptom level of the network and assess the projected effects of node‐specific interventions on the entire network. By systematically reducing or increasing the threshold parameters (i.e., intercept, or means the onset levels of symptoms) of the Ising network by two standard deviations, NIRA simulated symptom alleviation or aggravation, and then identified specific symptoms that had the greatest impact on the overall symptom levels (i.e., sum scores of the whole network structure). The NIRA outcomes were determined by the absolute difference between the network's sum scores before and after the interventions. In alleviating interventions, the symptoms that most effectively reduce the sum score are identified as the primary targets for interventions, and in aggravating interventions, those that most significantly increase the sum score are identified as the primary targets for prevention, respectively (Li et al. 2024; Lunansky et al. 2022). Given previous studies (Guo et al. 2025; Tao et al. 2024), we also conducted Mann–Whitney tests on the NIRA values to investigate whether males and females had different targets for simulated interventions.

## 3 Results

### 3.1 Descriptive Information

Table S1 shows descriptive statistics of continuous and binary variables for the total sample and by gender. Meanwhile, Table 1 reports the corresponding gender differences. Males and females did not differ in the total SNSATS score (*p* = 0.196). However, females reported higher levels of SNSATS6 (“virtual friend anxiety”; *p* = 0.002, *d* = 0.17) and marginally higher levels of SNSATS3 (“dual existence”; *p* = 0.069, *d* = 0.10). Conversely, males showed marginally higher levels of SNSATS5 (“online relationship satisfaction”; *p* = 0.085, *d* = 0.10). Males also exhibited higher levels on the total AOABS score (*p* < 0.001, *d* = 0.38) and all four sub‐aspects (*p*s < 0.001, *d*s ≥ 0.27). Results based on dichotomized data were largely consistent with those derived from the continuous measures (see details in Table 1).

**TABLE 1:** Descriptive information and *t*‐test results between genders.

Data	Variable	Gender differences				
		Mean (SD)		Diff	*p*	Cohen's *d*
		Male	Female			
Continuous	SNSATS	2.51 (0.87)	2.58 (0.82)	−0.06 ([−0.15, 0.03])	0.196	−0.07 ([−0.18, 0.04])
	SNSATS1	2.55 (1.05)	2.58 (0.98)	−0.03 ([−0.15, 0.08])	0.545	−0.03 ([−0.14, 0.08])
	SNSATS2^a^	2.82 (1.20)	2.90 (1.16)	−0.08 ([−0.21, 0.05])	0.217	−0.07 ([−0.18, 0.04])
	SNSATS3^a^	2.79 (1.22)	2.92 (1.21)	−0.12 ([−0.25, 0.01])	0.069	−0.10 ([−0.21, 0.01])
	SNSATS4^a^	2.40 (1.11)	2.45 (1.08)	−0.04 ([−0.16, 0.07])	0.458	−0.04 ([−0.15, 0.07])
	SNSATS5^a^	2.37 (1.09)	2.26 (1.09)	0.10 ([−0.01, 0.22])	0.084	0.10 ([−0.01, 0.20])
	SNSATS6	2.16 (1.05)	2.35 (1.11)	−0.19 ([−0.30, −0.07])	0.002	−0.17 ([−0.28, −0.06])
	AOABS	1.23 (0.36)	1.11 (0.25)	0.12 ([0.08, 0.15])	< 0.001	0.38 ([0.27, 0.49])
	AOABS1	1.29 (0.39)	1.14 (0.29)	0.15 ([0.12, 0.19])	< 0.001	0.46 ([0.35, 0.57])
	AOABS2	1.21 (0.37)	1.12 (0.28)	0.09 ([0.05, 0.13])	< 0.001	0.27 ([0.16, 0.38])
	AOABS3	1.25 (0.38)	1.10 (0.25)	0.15 ([0.11, 0.18])	< 0.001	0.45 ([0.34, 0.56])
	AOABS4	1.19 (0.39)	1.10 (0.25)	0.09 ([0.06, 0.13])	< 0.001	0.28 ([0.17, 0.39])
Binary	SNSATS					
	SNSATS1^a^	0.53 (0.50)	0.52 (0.50)	0.01 [−0.04, 0.06]	0.712	0.02 [−0.09, 0.13]
	SNSATS2^a^	0.59 (0.49)	0.60 (0.49)	−0.01 [−0.06, 0.04]	0.720	−0.02 [−0.13, 0.09]
	SNSATS3	0.60 (0.49)	0.64 (0.48)	−0.04 [−0.10, 0.01]	0.108	−0.09 [−0.20, 0.02]
	SNSATS4^a^	0.46 (0.50)	0.46 (0.50)	0.00 [−0.05, 0.06]	0.864	0.01 [−0.10, 0.12]
	SNSATS5	0.47 (0.50)	0.40 (0.49)	0.08 [0.02, 0.13]	0.006	0.15 [0.04, 0.26]
	SNSATS6	0.40 (0.49)	0.45 (0.50)	−0.06 [−0.11, −0.00]	0.041	−0.11 [−0.22, −0.00]
	AOABS					
	AOABS1	0.35 (0.48)	0.16 (0.37)	0.19 [0.15, 0.24]	< 0.001	0.45 [0.34, 0.56]
	AOABS2	0.25 (0.43)	0.17 (0.37)	0.08 [0.04, 0.13]	< 0.001	0.20 [0.09, 0.31]
	AOABS3	0.31 (0.46)	0.13 (0.34)	0.18 [0.13, 0.22]	< 0.001	0.43 [0.32, 0.54]
	AOABS4	0.22 (0.42)	0.14 (0.34)	0.09 [0.04, 0.13]	< 0.001	0.22 [0.11, 0.33]

Abbreviations: AOABS = Adolescent Online Aggressive Behavior Scale; AOABS1 = Instrumental overt aggression; AOABS2 = Instrumental relational aggression; AOABS3 = Reactive overt aggression; AOABS4 = Reactive relational aggression; SNSATS = Social Network Sites Addictive Tendencies Scale; SNSATS1 = Declining productivity; SNSATS2 = Insomnia; SNSATS3 = Dual existence; SNSATS4 = Encroach on other activities; SNSATS5 = Online relationship satisfaction; SNSATS6 = Virtual friend anxiety.

^a^ Except for variables marked with “(a)”, group differences were examined using Welch's *t*‐tests, as some variables violated the assumption of homogeneity of variance.

### 3.2 Network Structures and Centrality Indices

**FIGURE 1:**
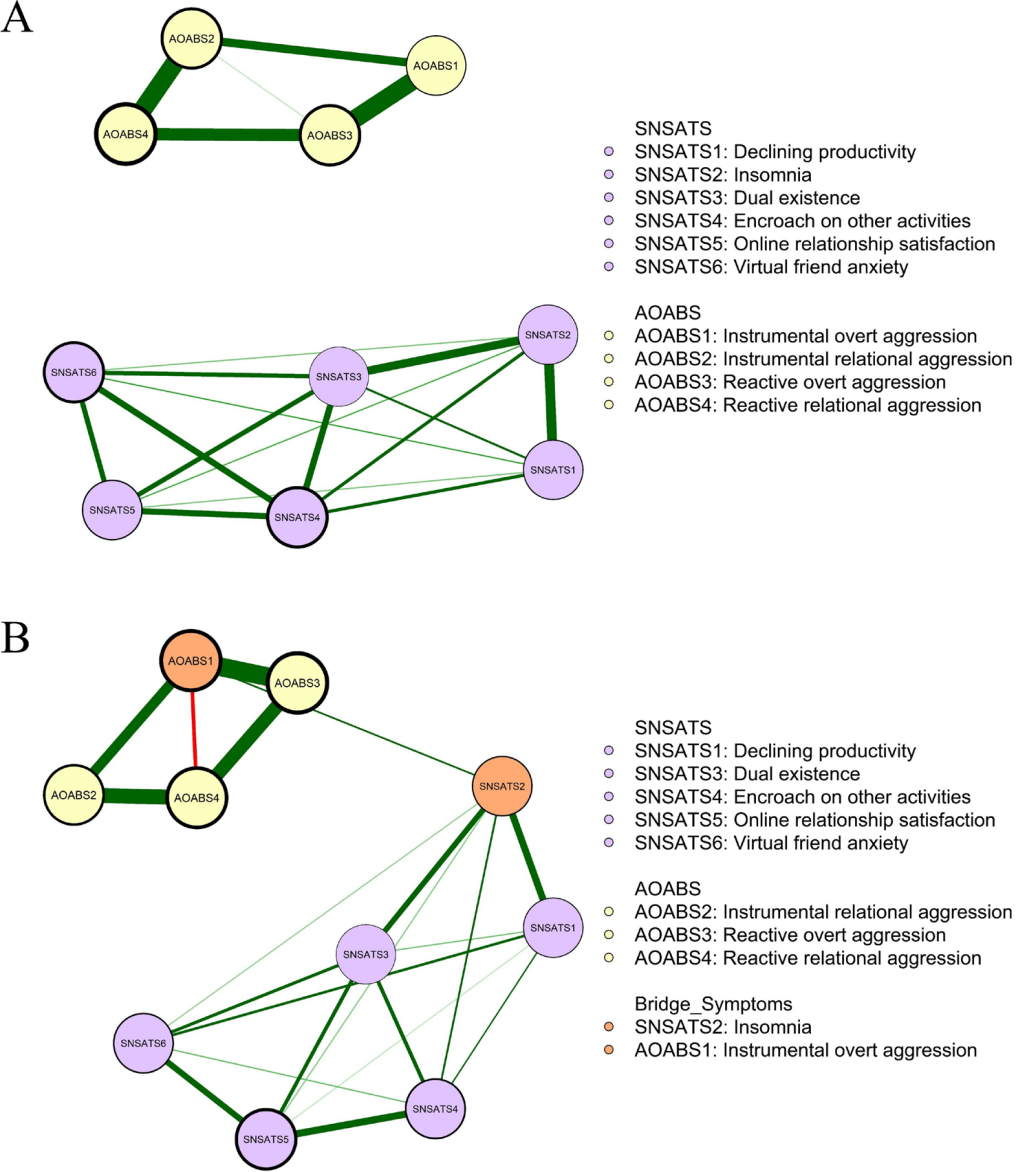
(A) Network structure for males (*n* = 580). (B) Network structure for females (*n* = 745). Positive associations between symptoms are denoted by green lines, while negative associations are represented by red lines. Edge thickness indicates association strength. The thickness of the node borders reflects the absolute values of the nodes' threshold parameters. All symptom thresholds suggest a tendency toward absence (as their negative threshold values).

**FIGURE 2:**
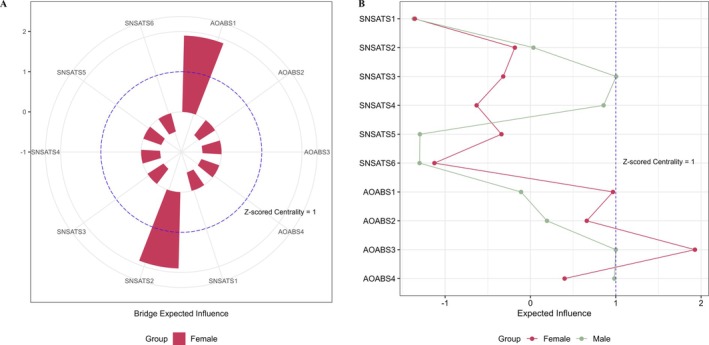
(A) The bridge EI values for nodes are in two groups. (B) The EI values for all nodes are in two groups.

Item‐check results indicated that all variables met the prerequisites for network analysis. Figure 1 illustrates the SNSATS‐AOABS Ising networks for males (*n* = 580) and females (*n* = 745), while Figure 2 provides the EI values and bridge EI values for all nodes. Detailed adjacency matrices, including edge weights, thresholds, and centrality indices (i.e., EI and bridge EI), are referenced in Tables S2 and S3. All nodes in both networks exhibited negative thresholds, suggesting that these factors tended to remain at a low level of activation. SNSATS3 (“dual existence”) had a threshold closest to zero in both the male (intercept = −1.817) and female (intercept = −1.305) networks, suggesting that it was the most easily triggered behavior.

The male network (Figure 1A) consisted of 20 non‐zero edges, all of which were positive. Social network addictive behaviors and online aggressive behaviors formed two separate clusters with no connecting edges. SNSATS3 (“dual existence”; EI = 1.004) and AOABS3 (“reactive overt aggression”; EI = 1.000) exhibited the highest EI centrality, marking them as core nodes (Figure 2B). SNSATS3 was strongly linked to five other SNSATS nodes (edge weights range from 0.649 to 1.666), particularly SNSATS2 (“insomnia”; edge weight = 1.666) and SNSATS4 (“encroach on other activities”; edge weight = 1.340). AOABS3 was strongly linked to AOABS1 (“instrumental overt aggression”; edge weight = 3.292) and AOABS4 (“reactive relational aggression”; edge weight = 2.327).

In the female network (Figure 1B), 21 non‐zero edges were detected; 20 were positive, and one negative edge emerged between AOABS1 (“instrumental overt aggression”) and AOABS4 (“reactive relational aggression”; edge weight = −1.005). In contrast to the male network, social network addictive behaviors and online aggressive behaviors formed two clusters connected via an edge between AOABS1 and SNSATS2 (“insomnia”; edge weight = 0.584). These nodes were therefore identified as bridging nodes (Bridge EIs = 1.897; Figure 2A). AOABS3 (“reactive overt aggression”; EI = 1.928; Figure 2B) exhibited the highest EI centrality, indicating its core role within the network structure. AOABS3 was strongly linked to AOABS1 (edge weight = 4.160) and AOABS4 (edge weight = 3.065).

To rule out the impact of dichotomization on network estimation, we constructed male and female networks using the original continuous data (Figure S1).

Mantel tests revealed a strong correlation between the edge‐weight matrices of the dichotomized and continuous networks in both males (*r* = 0.86, *p* = 0.001) and females (*r* = 0.87, *p* = 0.001). Therefore, dichotomized networks captured the underlying variable relations similarly to the continuous networks.

### 3.3 Network Comparison Test

The NCT indicated no significant differences in the *Ising* network structure (*M* = 1.02, *p* = 0.243, Bonferroni‐adjusted *p* = 0.243) or global strength (*S* = 1.65, *p* = 0.334, Bonferroni‐adjusted *p* = 0.334; see Figure S2) between males and females. The edge invariance test (Table S4) indicated that males exhibited stronger edges between SNSATS4 and SNSATS6 (“encroach on other activities‐virtual friend anxiety”; diff = 1.106, *p* = 0.004) and between AOABS2 and AOABS3 (“instrumental relational aggression‐reactive overt aggression”; diff = 0.110, *p* = 0.039). Females showed stronger edges between SNSATS2 and AOABS1 (“insomnia‐instrumental overt aggression”; diff = 0.584, *p* = 0.033) and between AOABS1 and AOABS4 (“instrumental overt aggression‐reactive relational aggression”; diff = 1.005, *p* = 0.026). Females were also marginally stronger in the edge between AOABS1 and AOABS3 (diff = 0.868, *p* = 0.075).

The centrality invariance test (Table S5) indicated that males had higher EI centrality for SNSATS3 (“dual existence”; diff = 1.133, *p* = 0.094; marginal) and SNSATS4 (diff = 1.397, *p* = 0.022; significant), whereas females had higher EI centrality for AOABS3 (diff = 1.496, *p* = 0.046; significant). Females also had higher bridge EI for SNSATS2 (“insomnia”; diff = 0.584, *p* = 0.057) and AOABS1 (“instrumental overt aggression”; diff = 0.584, *p* = 0.099). However, after Bonferroni correction, none of the differences in the edge weights (adjusted *p*s ≥ 0.180), EI centrality (adjusted *p*s ≥ 0.440), or bridge EI centrality (adjusted *p*s = 1.000) remained significant. Figure S2 also presents additional analyses of network differences between the two groups using continuous data.

### 3.4 Network Accuracy and Stability

Figure S3 displays the results of the bootstrap analysis for edge weights in male and female networks. In general, the 95% CIs were adequately narrow, indicating stable results. Figure S4 shows that the CS‐Cs of EI were 0.44 for males and 0.52 for females, both exceeding the acceptable threshold of 0.25 and thus indicating sufficient stability. In contrast, the bridge EI values were unstable for both males and females (CS‐Cs < 0.25), likely due to the presence of only one weak edge linking the SNSATS and AOABS nodes. As most nodes had a bridge EI of zero, overall stability was low, and therefore, these results should be interpreted with caution. Figure S5 presents the results of the bootstrapped difference tests for the edge weights, for both genders. In general, several edges were significantly stronger than other edges in both networks. However, the edges linking the SNSATS and AOABS nodes in the female network (i.e., SNSATS2‐AOABS1) were relatively weaker and should be interpreted with greater caution. For both genders, bootstrapped difference tests on EI (Figure S6) and bridge EI (Figure S7) also indicated that the rankings of centrality were not robust and should be interpreted with caution. Only a few nodes showed differences in EI in both networks, and no nodes differed in bridge EI.

### 3.5 Simulation of Alleviating and Aggravating Interventions for Groups

**FIGURE 3:**
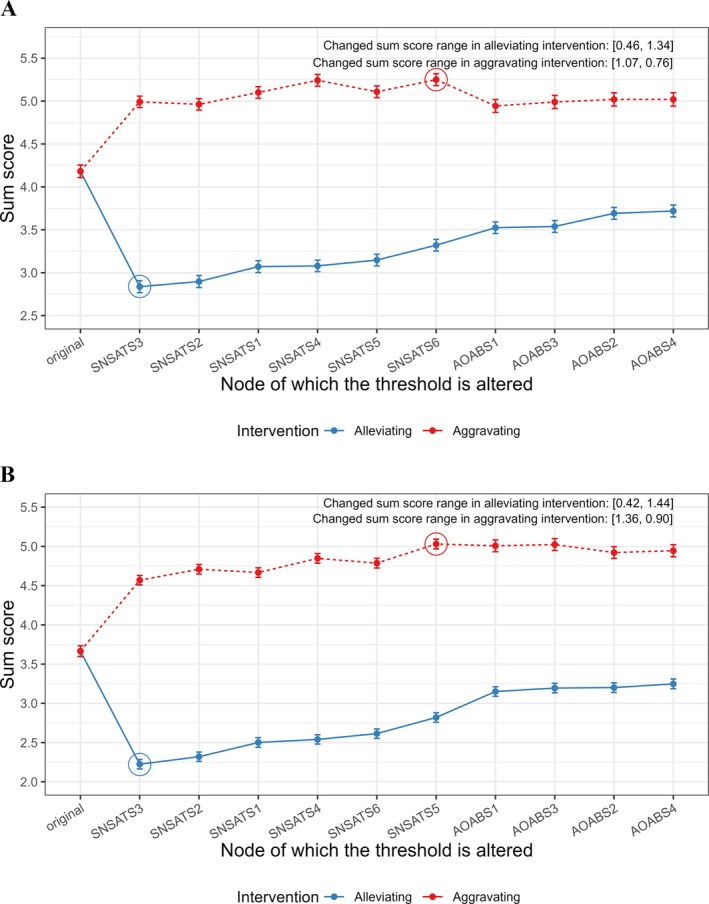
(A) Simulated alleviating intervention for males. (B) Dots represent the network sum scores, while the lines represent the 95% confidence intervals. The symptoms are listed based on the size of the intervention's effects, along with the original total score before the intervention.

Figure 3 and Table 2 display the results of simulated interventions for male and female networks. Alleviating interventions targeting SNSATS3 (“Dual existence”) could significantly further decrease overall symptom levels for both males (from 4.18 to 2.84, NIRA = 1.34; Figure 3A) and females (from 3.67 to 2.22, NIRA = 1.44; Figure 3B). These results suggested that this node could be a promising target for alleviating overall symptoms in both groups. Moreover, aggravating interventions targeting SNSATS6 (“virtual friend anxiety”) further increase the overall symptom levels for males (from 4.18 to 5.25, NIRA = 1.07; Figure 3A), and interventions targeting SNSATS5 (“online relationship satisfaction”) had a similar effect for females (from 3.67 to 5.03, NIRA = 1.37; Figure 3B). These findings indicated that these nodes may serve as suitable targets for mitigating the escalation of overall symptoms in the SNSATS‐AOABS network for each group.

**TABLE 2:** The results of simulated weakening and enhancing interventions between gender groups.

Group	Symptom	Original sum score	Alleviating intervention		Aggravating intervention	
			Sum score	NIRA	Sum score	NIRA
Males	SNSATS1	4.18	3.07	1.11	5.10	0.92
	SNSATS2	4.18	2.90	1.28	4.96	0.78
	SNSATS3	4.18	2.84	**1.34**	4.99	0.81
	SNSATS4	4.18	3.08	1.10	5.24	1.06
	SNSATS5	4.18	3.15	1.03	5.11	0.93
	SNSATS6	4.18	3.32	0.86	5.25	**1.07**
	AOABS1	4.18	3.52	0.66	4.94	0.76
	AOABS2	4.18	3.69	0.49	5.02	0.84
	AOABS3	4.18	3.54	0.64	4.99	0.81
	AOABS4	4.18	3.72	0.46	5.02	0.84
Females	SNSATS1	3.67	2.50	1.16	4.67	1.00
	SNSATS2	3.67	2.32	1.35	4.71	1.04
	SNSATS3	3.67	2.22	**1.44**	4.57	0.90
	SNSATS4	3.67	2.54	1.13	4.85	1.18
	SNSATS5	3.67	2.82	0.85	5.03	**1.37**
	SNSATS6	3.67	2.61	1.05	4.79	1.12
	AOABS1	3.67	3.15	0.51	5.01	1.34
	AOABS2	3.67	3.20	0.46	4.92	1.26
	AOABS3	3.67	3.19	0.47	5.02	1.36
	AOABS4	3.67	3.25	0.42	4.94	1.28

*Note:* The highest NIRA scores were highlighted in bold.

To explore the gender differences in the simulated interventions, we compared the NIRA values of alleviating and aggravating interventions between males and females. Mann–Whitney tests indicated that males and females did not differ in the NIRA outcomes of the alleviating intervention (*W* = 50.00, *p* = 1.000), but did differ in the NIRA outcomes of the aggravating intervention (*W* = 8.00, *p* = 0.001).

## 4 Discussion

The interrelation between problematic SNS use and online aggression can hardly be ignored in contemporary society. The present study aimed to address the problem by examining the underlying mechanism of the link between the two variables from a network analysis perspective. Furthermore, potential targets for intervention and prevention were presented through simulations that illustrated the alleviation and aggravation of symptoms. Some findings are worth discussing.

First, it should be noted that this study examined gender differences using both continuous and dichotomized scoring approaches. Findings indicated that for “online relationship satisfaction”, males' continuous score was marginally higher than females' (*p* = 0.085), whereas the dichotomized scoring method yielded statistically significant differences (*p* = 0.006). Results for all other scores remained largely consistent across both analytical approaches. Furthermore, it is also important to emphasize that the analysis, using both continuous and binary scores, revealed no bridge symptoms that connected problematic SNS use with online aggression in the male group. In the female group, despite the continuous network identifying two bridge symptoms and the dichotomous network only one, this discrepancy did not result in significant structural variations within the network. Taken together, these findings suggest that the dichotomized networks captured the underlying relationships similarly to the continuous ones, and the choice of scoring method does not substantially alter the overall conclusions of the study, though interpretations of specific outcomes may require some caution. Following these findings, an ensuing discussion will draw upon the results from the dichotomized data.

The absence of bridging symptoms between these two behaviors in the male group is a noteworthy and unexpected finding. Prior research has consistently documented associations between SNS usage time and aggressive behavior (Chan et al. 2019; Darazi et al. 2023; Erdur‐Baker 2010). Dehue et al. (2008) further found that males were more likely to commit overt cyber aggression (i.e., hacking) through SNSs. The results of other studies also showed perpetrators of online harassment were usually young males (Erdur‐Baker 2010). However, this study identifies a distinct pattern in which the connection between SNS use and online aggression appears absent, suggesting that the two behaviors among males may constitute two functionally independent behavioral constructs. We speculate that PSNS among male college students is predominantly driven by immersive engagement, achievement‐oriented pursuits, and task‐focused motivations, such as gaming, rather than by high‐intensity interpersonal interaction or social comparison (Kuss and Griffiths 2012; Wu et al. 2013). Such a pattern of use inherently generates less social friction and therefore triggers less hostile expression or confrontational behavior. Furthermore, prior research suggests that men are more inclined to adopt avoidance‐based strategies in emotion regulation, instead of expressing negative affect through interpersonal channels (Kardefelt‐Winther 2014). Given that online aggression is typically associated with anger expression and retaliatory motives, this attenuated “emotion to aggression” pathway among males may explain the absence of bridge symptoms between PSNS and online aggression.

In terms of female network structure, results from this study indicated clearly that their excessive SNS use is only associated with instrumental aggression and not reactive aggression, as “insomnia” interacted with explicit “instrumental overt aggression”. This finding is partially consistent with the results demonstrated by Osgood et al. (2021), who suggested that subjective sleep was associated with perpetrating relational aggression at work. The “insomnia” symptom of PSNS refers to the compressed sleep time, which has been proven to be associated with subsequent poor emotion regulation (Palmer and Alfano 2017). In addition, according to the social role theory (Eagly et al. 2000), females should be obedient and avoid conflicts with others even when attacked. However, this gender constraint may be weakened when transferred to the online world, as SNSs provide anonymous settings and the possibility of attacking others without being caught and punished (Wen et al. 2023), or a lack of self‐control at a lower level (Lu et al. 2025). Therefore, we suspected that this connection could also be attributed to females' suppressed negative emotions caused by the lack of sleep being released online, leading them to exhibit more overt and direct forms of aggression. In summary, the findings of this study suggest that females who experience insufficient sleep are more likely to engage in proactive aggressive behaviors.

As for the simulation intervention results, “dual existence” could be the effective intervention target for both groups, and “virtual friend anxiety” and “online relationship satisfaction” may be the effective prevention targets for males and females, respectively. “Dual existence” is seen as a disconnect between the online world and the real world. The alleviation effect this symptom has on the overall network can be explained by the fact that when the sense of separation between virtual and reality is reduced, perhaps individual online aggressive behavior will decrease. The findings also showed the particularly critical preventative role of “virtual friend anxiety” in shaping males' PSNS and OAB. Heightened virtual friend anxiety reflects perceived insufficiency or instability in online social relationships, which can amplify negative affect and social frustration. Consistent with prior research (Zhang et al. 2022), showing that individuals with elevated levels of social dissatisfaction or loneliness are prone to use social media excessively, often as a compensatory strategy, and are more likely to engage in hostile or aggressive online behavior. For females, “online relationship satisfaction”, which reflects the reliance on virtual interpersonal relationships on SNSs, becomes the key preventative target. Indeed, a user embedded in a virtual network wants to enhance their social interaction, receive social support (Wu et al. 2024), and establish new relationships with others embedded in the SNS networks (Manago et al. 2012). Lin and Lu (2011) also found that the number of peers is an important factor affecting the continued intention to use for females but not for males. The significant effect of this symptom implies that the companionship provided by online friends may provide more than just enjoyment for girls, and those virtual relationships also increase the risk of random aggression against others online.

## 5 Limitations

This present study also has some limitations that should be noted. First, although the cross‐sectional design employed in this study enables investigation of which symptoms have the greatest influence on the network structure of PSUS use and OAB, the results regarding the centrality of symptoms that link the two met the criterion as expected, but were not robust enough. Therefore, future research should consider adopting a longitudinal design or multiple central datasets (Xu et al. 2026) to strengthen the robustness of the research outcomes. Second, the study was conducted online, and all data were self‐reported and collected from a single province, suggesting that participant biases and regional differences may have affected the accuracy of the results. For instance, participants may intentionally underreport their online aggressive behaviors to maintain a positive self‐image, which could attenuate the strength of associations between symptoms in network analysis, thereby potentially obscuring the true structure of the symptom network. This may partly explain the low edge weight accuracy and unstable centrality estimates in our networks. More objective measurement methods and larger sample size studies should be considered in future research. This would also facilitate the detection of more subtle and stable gender differences in network structures, thereby replicating and extending our findings. Finally, our respondents were general SNS users, and we did not focus on any specific SNS applications. This methodological choice may constrain a nuanced understanding of platform‐specific dynamics, suggesting that future research could focus on one certain SNS platform as a specialized research direction.

## 6 Conclusions

The current study revealed significant between‐group differences in symptom associations. Specifically, while no significant connections were observed between PSNS and OAB in the male group, in the female group, symptoms of “insomnia” and “instrumental overt aggression” were found to bridge the association between these two conditions. Besides, “encroach on other activities” may be the most effective target for intervention for both groups, but “virtual friend anxiety” and “online relationship satisfaction” should be considered separately and cautiously for preventive care when dealing with males and females. These results have an enlightening effect on the treatment of PSNS and OAB among students of different genders.

## Funding

The current study was supported by Research Grant from “National Center for Mental Health, China‐East China Normal University” Mental Health Digital Governance Innovation Institute.

## Ethics Statement

This research was examined and approved by the ethics committee of Beijing Normal University (Reference number: 202305290090). Informed consent was obtained from all individual participants included in the study.

## Consent

Informed consent was obtained from all individual participants included in the study.

## Conflicts of Interest

The authors declare no conflicts of interest.

## Supporting information


**Data S1:** Supporting Information.

## Data Availability

The data that support the findings of this study are available on request from the corresponding author. The data are not publicly available due to privacy or ethical restrictions.
