# Supplementary material for: Simulating Interventions for Symptoms Linking Problematic Social Networking Sites Use to Online Aggressive Behavior Among Chinese College Students: A Gender‐Differentiated Network Analysis
Source: Psych J. 2026 Mar 1;15(2):e70085. doi: 10.1002/pchj.70085 (PMC12950332; doi:10.1002/pchj.70085)
Supplement: Supplementary file 1 — Data S1: Supporting Information. [file PCHJ-15-e70085-s001.docx]

**Online Supplemental Materials**

**Tables**

**Table S1. Descriptive statistics of study variables for the total sample and by gender.**

| **Sample** |  | **Variable** | **Continuous score** | | | |  | **Binary score** | | | |
| --- | --- | --- | --- | --- | --- | --- | --- | --- | --- | --- | --- |
|  |  |  | ***Mean* (*SD*)** | ***Skewness*** | ***Kurtosis*** | ***Median* (*IQR*)** |  | ***Mean* (*SD*)** | ***Skewness*** | ***Kurtosis*** | ***Median* (*IQR*)** |
| **Total** |  | **SNSATS** | 2.55 (0.84) | -0.07 | -0.29 | 2.67 (1.00) |  |  |  |  |  |
| **(*N* = 1325)** |  | SNSATS1 | 2.57 (1.01) | 0.15 | -0.58 | 3.00 (1.00) |  | 0.53 (0.50) | -0.10 | -1.99 | 1.00 (1.00) |
|  |  | SNSATS2 | 2.86 (1.18) | -0.02 | -1.02 | 3.00 (2.00) |  | 0.59 (0.49) | -0.37 | -1.86 | 1.00 (1.00) |
|  |  | SNSATS3 | 2.86 (1.22) | -0.07 | -1.00 | 3.00 (2.00) |  | 0.62 (0.48) | -0.51 | -1.75 | 1.00 (1.00) |
|  |  | SNSATS4 | 2.43 (1.09) | 0.36 | -0.60 | 2.00 (1.00) |  | 0.46 (0.50) | 0.16 | -1.98 | 0.00 (1.00) |
|  |  | SNSATS5 | 2.31 (1.09) | 0.46 | -0.47 | 2.00 (2.00) |  | 0.43 (0.50) | 0.28 | -1.93 | 0.00 (1.00) |
|  |  | SNSATS6 | 2.27 (1.09) | 0.41 | -0.70 | 2.00 (2.00) |  | 0.43 (0.49) | 0.29 | -1.92 | 0.00 (1.00) |
|  |  | **AOABS** | 1.17 (0.31) | 2.47 | 5.83 | 1.03 (0.13) |  |  |  |  |  |
|  |  | AOABS1 | 1.20 (0.34) | 2.20 | 4.97 | 1.00 (0.14) |  | 0.25 (0.43) | 1.18 | -0.60 | 0.00 (0.00) |
|  |  | AOABS2 | 1.16 (0.33) | 2.63 | 7.52 | 1.00 (0.12) |  | 0.20 (0.40) | 1.48 | 0.19 | 0.00 (0.00) |
|  |  | AOABS3 | 1.17 (0.32) | 2.28 | 4.73 | 1.00 (0.12) |  | 0.21 (0.41) | 1.41 | 0.00 | 0.00 (0.00) |
|  |  | AOABS4 | 1.14 (0.32) | 2.68 | 7.29 | 1.00 (0.12) |  | 0.17 (0.38) | 1.73 | 0.99 | 0.00 (0.00) |
| **Male** |  | **SNSATS** | 2.51 (0.87) | -0.06 | -0.37 | 2.67 (1.00) |  |  |  |  |  |
| **(*n* = 580)** |  | SNSATS1 | 2.55 (1.05) | 0.17 | -0.60 | 3.00 (1.00) |  | 0.53 (0.50) | -0.12 | -1.99 | 1.00 (1.00) |
|  |  | SNSATS2 | 2.82 (1.20) | 0.03 | -1.01 | 3.00 (2.00) |  | 0.59 (0.49) | -0.35 | -1.88 | 1.00 (1.00) |
|  |  | SNSATS3 | 2.79 (1.22) | 0.02 | -0.99 | 3.00 (2.00) |  | 0.60 (0.49) | -0.40 | -1.84 | 1.00 (1.00) |
|  |  | SNSATS4 | 2.40 (1.11) | 0.40 | -0.55 | 2.00 (2.00) |  | 0.46 (0.50) | 0.14 | -1.98 | 0.00 (1.00) |
|  |  | SNSATS5 | 2.37 (1.09) | 0.37 | -0.48 | 2.00 (2.00) |  | 0.47 (0.50) | 0.10 | -1.99 | 0.00 (1.00) |
|  |  | SNSATS6 | 2.16 (1.05) | 0.48 | -0.59 | 2.00 (2.00) |  | 0.40 (0.49) | 0.42 | -1.83 | 0.00 (1.00) |
|  |  | **AOABS** | 1.23 (0.36) | 1.89 | 3.04 | 1.06 (0.23) |  |  |  |  |  |
|  |  | AOABS1 | 1.29 (0.39) | 1.58 | 2.08 | 1.14 (0.43) |  | 0.35 (0.48) | 0.61 | -1.63 | 0.00 (1.00) |
|  |  | AOABS2 | 1.21 (0.37) | 1.95 | 3.12 | 1.00 (0.12) |  | 0.25 (0.43) | 1.16 | -0.65 | 0.00 (0.00) |
|  |  | AOABS3 | 1.25 (0.38) | 1.70 | 2.26 | 1.12 (0.25) |  | 0.31 (0.46) | 0.82 | -1.33 | 0.00 (1.00) |
|  |  | AOABS4 | 1.19 (0.39) | 2.14 | 4.32 | 1.00 (0.12) |  | 0.22 (0.42) | 1.34 | -0.20 | 0.00 (0.00) |
| **Female** |  | **SNSATS** | 2.58 (0.82) | -0.07 | -0.25 | 2.67 (1.00) |  |  |  |  |  |
| **(*n* = 745)** |  | SNSATS1 | 2.58 (0.98) | 0.15 | -0.59 | 3.00 (1.00) |  | 0.52 (0.50) | -0.08 | -2.00 | 1.00 (1.00) |
|  |  | SNSATS2 | 2.90 (1.16) | -0.05 | -1.03 | 3.00 (2.00) |  | 0.60 (0.49) | -0.39 | -1.85 | 1.00 (1.00) |
|  |  | SNSATS3 | 2.92 (1.21) | -0.15 | -0.99 | 3.00 (2.00) |  | 0.64 (0.48) | -0.59 | -1.65 | 1.00 (1.00) |
|  |  | SNSATS4 | 2.45 (1.08) | 0.34 | -0.65 | 2.00 (1.00) |  | 0.46 (0.50) | 0.16 | -1.98 | 0.00 (1.00) |
|  |  | SNSATS5 | 2.26 (1.09) | 0.53 | -0.45 | 2.00 (2.00) |  | 0.40 (0.49) | 0.41 | -1.83 | 0.00 (1.00) |
|  |  | SNSATS6 | 2.35 (1.11) | 0.34 | -0.79 | 2.00 (2.00) |  | 0.45 (0.50) | 0.19 | -1.97 | 0.00 (1.00) |
|  |  | **AOABS** | 1.11 (0.25) | 3.19 | 10.20 | 1.00 (0.10) |  |  |  |  |  |
|  |  | AOABS1 | 1.14 (0.29) | 3.06 | 10.98 | 1.00 (0.14) |  | 0.16 (0.37) | 1.84 | 1.39 | 0.00 (0.00) |
|  |  | AOABS2 | 1.12 (0.28) | 3.53 | 15.91 | 1.00 (0.12) |  | 0.17 (0.37) | 1.79 | 1.20 | 0.00 (0.00) |
|  |  | AOABS3 | 1.10 (0.25) | 2.99 | 8.33 | 1.00 (0.12) |  | 0.13 (0.34) | 2.14 | 2.59 | 0.00 (0.00) |
|  |  | AOABS4 | 1.10 (0.25) | 3.22 | 10.39 | 1.00 (0.00) |  | 0.14 (0.34) | 2.12 | 2.52 | 0.00 (0.00) |

***Note.*** SD = standard deviation; IQR = interquartile range, representing the difference between the 75th and 25th percentiles (Q3 − Q1). SNSATS = Social Network Sites Addictive Tendencies Scale: SNSATS1 = Declining productivity, SNSATS2 = Insomnia, SNSATS3 = Dual existence, SNSATS4 = Encroach on other activities, SNSATS5 = Online relationship satisfaction, SNSATS6 = Virtual friend anxiety; AOABS = Adolescent Online Aggressive Behavior Scale: AOABS1 = Instrumental overt aggression, AOABS2 = Instrumental relational aggression, AOABS3 = Reactive overt aggression, AOABS4 = Reactive relational aggression.

**Table S2.** Weighted adjacency matrix of SNSATS and AOABS for males.

|  | Edge-weights matrix | | | | | | | | | |  | Threshold |  | Standardized  Centrality | |
| --- | --- | --- | --- | --- | --- | --- | --- | --- | --- | --- | --- | --- | --- | --- | --- |
|  | SNSATS1 | SNSATS2 | SNSATS3 | SNSATS4 | SNSATS5 | SNSATS6 | AOABS1 | AOABS2 | AOABS3 | AOABS4 |  |  |  | EI | Bridge EI |
| **SNSATS** |  |  |  |  |  |  |  |  |  |  |  |  |  |  |  |
| SNSATS1: Declining productivity | 0.000 | 1.912 | 0.649 | 0.842 | 0.268 | 0.435 | 0.000 | 0.000 | 0.000 | 0.000 |  | -2.078 |  | -1.367 | —— |
| SNSATS2: Insomnia | 1.912 | 0.000 | 1.666 | 0.834 | 0.383 | 0.272 | 0.000 | 0.000 | 0.000 | 0.000 |  | -1.989 |  | 0.035 | —— |
| SNSATS3: Dual existence | 0.649 | 1.666 | 0.000 | 1.340 | 1.081 | 0.995 | 0.000 | 0.000 | 0.000 | 0.000 |  | **-1.817** |  | **1.004** | —— |
| SNSATS4: Encroach on other activities | 0.842 | 0.834 | 1.340 | 0.000 | 1.294 | 1.322 | 0.000 | 0.000 | 0.000 | 0.000 |  | -3.268 |  | 0.857 | —— |
| SNSATS5: Online relationship satisfaction | 0.268 | 0.383 | 1.081 | 1.294 | 0.000 | 1.129 | 0.000 | 0.000 | 0.000 | 0.000 |  | -2.230 |  | -1.296 | —— |
| SNSATS6: Virtual friend anxiety | 0.435 | 0.272 | 0.995 | 1.322 | 1.129 | 0.000 | 0.000 | 0.000 | 0.000 | 0.000 |  | -3.082 |  | -1.300 | —— |
| **AOABS** |  |  |  |  |  |  |  |  |  |  |  |  |  |  |  |
| AOABS1: Instrumental overt aggression | 0.000 | 0.000 | 0.000 | 0.000 | 0.000 | 0.000 | 0.000 | 1.676 | 3.292 | 0.000 |  | -2.113 |  | -0.110 | —— |
| AOABS2: Instrumental relational aggression | 0.000 | 0.000 | 0.000 | 0.000 | 0.000 | 0.000 | 1.676 | 0.000 | 0.110 | 3.390 |  | -3.119 |  | 0.193 | —— |
| AOABS3: Reactive overt aggression | 0.000 | 0.000 | 0.000 | 0.000 | 0.000 | 0.000 | 3.292 | 0.110 | 0.000 | 2.327 |  | -3.170 |  | **1.000** | —— |
| AOABS4: Reactive relational aggression | 0.000 | 0.000 | 0.000 | 0.000 | 0.000 | 0.000 | 0.000 | 3.390 | 2.327 | 0.000 |  | -3.834 |  | 0.983 | —— |

**Table S3.** Weighted adjacency matrix of **SNSATS** and AOABS for females.

|  | Edge-weights matrix | | | | | | | | | |  | Threshold |  | Standardized Centrality | |
| --- | --- | --- | --- | --- | --- | --- | --- | --- | --- | --- | --- | --- | --- | --- | --- |
|  | SNSATS1 | SNSATS2 | SNSATS3 | SNSATS4 | SNSATS5 | SNSATS6 | AOABS1 | AOABS2 | AOABS3 | AOABS4 |  |  |  | EI | Bridge EI |
| **SNSATS** |  |  |  |  |  |  |  |  |  |  |  |  |  |  |  |
| SNSATS1 | 0.000 | 1.700 | 0.282 | 0.527 | 0.110 | 0.770 | 0.000 | 0.000 | 0.000 | 0.000 |  | -1.629 |  | -1.355 | -0.474 |
| SNSATS2 | 1.700 | 0.000 | 1.374 | 0.654 | 0.237 | 0.210 | 0.584 | 0.000 | 0.000 | 0.000 |  | -1.883 |  | -0.183 | 1.897 |
| SNSATS3 | 0.282 | 1.374 | 0.000 | 1.028 | 1.027 | 0.888 | 0.000 | 0.000 | 0.000 | 0.000 |  | **-1.305** |  | -0.319 | -0.474 |
| SNSATS4 | 0.527 | 0.654 | 1.028 | 0.000 | 1.720 | 0.306 | 0.000 | 0.000 | 0.000 | 0.000 |  | -2.237 |  | -0.631 | -0.474 |
| SNSATS5 | 0.110 | 0.237 | 1.027 | 1.720 | 0.000 | 1.482 | 0.000 | 0.000 | 0.000 | 0.000 |  | -3.406 |  | -0.340 | -0.474 |
| SNSATS6 | 0.770 | 0.210 | 0.888 | 0.306 | 1.482 | 0.000 | 0.000 | 0.000 | 0.000 | 0.000 |  | -2.016 |  | -1.126 | -0.474 |
| **AOABS** |  |  |  |  |  |  |  |  |  |  |  |  |  |  |  |
| AOABS1 | 0.000 | 0.584 | 0.000 | 0.000 | 0.000 | 0.000 | 0.000 | 2.364 | 4.160 | -1.005 |  | -3.875 |  | 0.967 | 1.897 |
| AOABS2 | 0.000 | 0.000 | 0.000 | 0.000 | 0.000 | 0.000 | 2.364 | 0.000 | 0.000 | 3.379 |  | -2.860 |  | 0.660 | -0.474 |
| AOABS3 | 0.000 | 0.000 | 0.000 | 0.000 | 0.000 | 0.000 | 4.160 | 0.000 | 0.000 | 3.065 |  | -4.076 |  | 1.928 | -0.474 |
| AOABS4 | 0.000 | 0.000 | 0.000 | 0.000 | 0.000 | 0.000 | -1.005 | 3.379 | 3.065 | 0.000 |  | -3.700 |  | 0.400 | -0.474 |

**Table S4: The differences in the edge-weights of networks between groups.**

| **Node1** | **Node2** | **Edge weight** | | | | ***difference*** | ***p*** | | **Bonferroni-adjusted *p*** | |
| --- | --- | --- | --- | --- | --- | --- | --- | --- | --- | --- |
|  |  | **Male** | | **Female** | |  |  |  |  |  |
| SNSATS1 | SNSATS2 | 1.912 |  | 1.700 |  | 0.213 | .523 |  | 1.000 |  |
| SNSATS1 | SNSATS3 | 0.649 |  | 0.282 |  | 0.367 | .295 |  | 1.000 |  |
| SNSATS2 | SNSATS3 | 1.666 |  | 1.374 |  | 0.292 | .433 |  | 1.000 |  |
| SNSATS1 | SNSATS4 | 0.842 |  | 0.527 |  | 0.315 | .407 |  | 1.000 |  |
| SNSATS2 | SNSATS4 | 0.834 |  | 0.654 |  | 0.180 | .620 |  | 1.000 |  |
| SNSATS3 | SNSATS4 | 1.340 |  | 1.028 |  | 0.312 | .471 |  | 1.000 |  |
| SNSATS1 | SNSATS5 | 0.268 |  | 0.110 |  | 0.159 | .738 |  | 1.000 |  |
| SNSATS2 | SNSATS5 | 0.383 |  | 0.237 |  | 0.147 | .706 |  | 1.000 |  |
| SNSATS3 | SNSATS5 | 1.081 |  | 1.027 |  | 0.055 | .893 |  | 1.000 |  |
| SNSATS4 | SNSATS5 | 1.294 |  | 1.720 |  | 0.426 | .183 |  | 1.000 |  |
| SNSATS1 | SNSATS6 | 0.435 |  | 0.770 |  | 0.335 | .339 |  | 1.000 |  |
| SNSATS2 | SNSATS6 | 0.272 |  | 0.210 |  | 0.062 | .886 |  | 1.000 |  |
| SNSATS3 | SNSATS6 | 0.995 |  | 0.888 |  | 0.107 | .792 |  | 1.000 |  |
| SNSATS4 | SNSATS6 | 1.322 |  | 0.306 |  | 1.016 | .004 | ** | .180 |  |
| SNSATS5 | SNSATS6 | 1.129 |  | 1.482 |  | 0.353 | .287 |  | 1.000 |  |
| SNSATS2 | AOABS1 | 0 |  | 0.584 |  | 0.584 | .033 | * | .438 |  |
| AOABS1 | AOABS2 | 1.676 |  | 2.364 |  | 0.687 | .115 |  | .862 |  |
| AOABS1 | AOABS3 | 3.292 |  | 4.160 |  | 0.868 | .075 | **†** | .674 |  |
| AOABS2 | AOABS3 | 0.110 |  | 0 |  | 0.110 | .039 | * | .438 |  |
| AOABS1 | AOABS4 | 0 |  | -1.005 |  | 1.005 | .026 | * | .438 |  |
| AOABS2 | AOABS4 | 3.390 |  | 3.379 |  | 0.011 | .976 |  | 1.000 |  |
| AOABS3 | AOABS4 | 2.327 |  | 3.065 |  | 0.737 | .155 |  | .995 |  |

***Note.*** ****, *p* < .01, * *p* < .05, **†** *p* < .1.

**Table S5: The differences in the EI and bridge EI of networks between groups.**

| **Centrality** |  | **Node** | **Raw value** | | **Difference** | | ***p*** | | **Bonferroni-adjusted *p*** | |
| --- | --- | --- | --- | --- | --- | --- | --- | --- | --- | --- |
|  |  |  | **Male** | **Female** |  |  |  |  |  |  |
| **EI** |  | **SNSATS** |  |  |  |  |  |  |  |  |
|  |  | SNSATS1 | 4.106 | 3.388 | 0.718 |  | .168 |  | 1.000 |  |
|  |  | SNSATS2 | 5.067 | 4.758 | 0.310 |  | .646 |  | 1.000 |  |
|  |  | SNSATS3 | 5.732 | 4.599 | 1.133 |  | .094 | **†** | 1.000 |  |
|  |  | SNSATS4 | 5.631 | 4.234 | 1.397 |  | .022 | ***** | .440 |  |
|  |  | SNSATS5 | 4.155 | 4.575 | -0.419 |  | .517 |  | 1.000 |  |
|  |  | SNSATS6 | 4.153 | 3.656 | 0.496 |  | .303 |  | 1.000 |  |
|  |  | **AOABS** |  |  |  |  |  |  |  |  |
|  |  | AOABS1 | 4.968 | 6.102 | -1.134 |  | .126 |  | 1.000 |  |
|  |  | AOABS2 | 5.176 | 5.743 | -0.567 |  | .463 |  | 1.000 |  |
|  |  | AOABS3 | 5.729 | 7.225 | -1.496 |  | .046 | * | .919 |  |
|  |  | AOABS4 | 5.718 | 5.440 | 0.278 |  | .688 |  | 1.000 |  |
| **Bridge EI** |  | **SNSATS** |  |  |  |  |  |  |  |  |
|  |  | SNSATS1 | 0 | 0 | 0 |  | 1.000 |  | 1.000 |  |
|  |  | SNSATS2 | 0 | 0.584 | -0.584 |  | .057 | **†** | 1.000 |  |
|  |  | SNSATS3 | 0 | 0 | 0 |  | 1.000 |  | 1.000 |  |
|  |  | SNSATS4 | 0 | 0 | 0 |  | 1.000 |  | 1.000 |  |
|  |  | SNSATS5 | 0 | 0 | 0 |  | 1.000 |  | 1.000 |  |
|  |  | SNSATS6 | 0 | 0 | 0 |  | 1.000 |  | 1.000 |  |
|  |  | **AOABS** |  |  |  |  |  |  |  |  |
|  |  | AOABS1 | 0 | 0.584 | -0.584 |  | .099 | **†** | 1.000 |  |
|  |  | AOABS2 | 0 | 0 | 0 |  | 1.000 |  | 1.000 |  |
|  |  | AOABS3 | 0 | 0 | 0 |  | 1.000 |  | 1.000 |  |
|  |  | AOABS4 | 0 | 0 | 0 |  | 1.000 |  | 1.000 |  |

***Note.*** * *p* < .05, **†** *p* < .1.

**Figures**


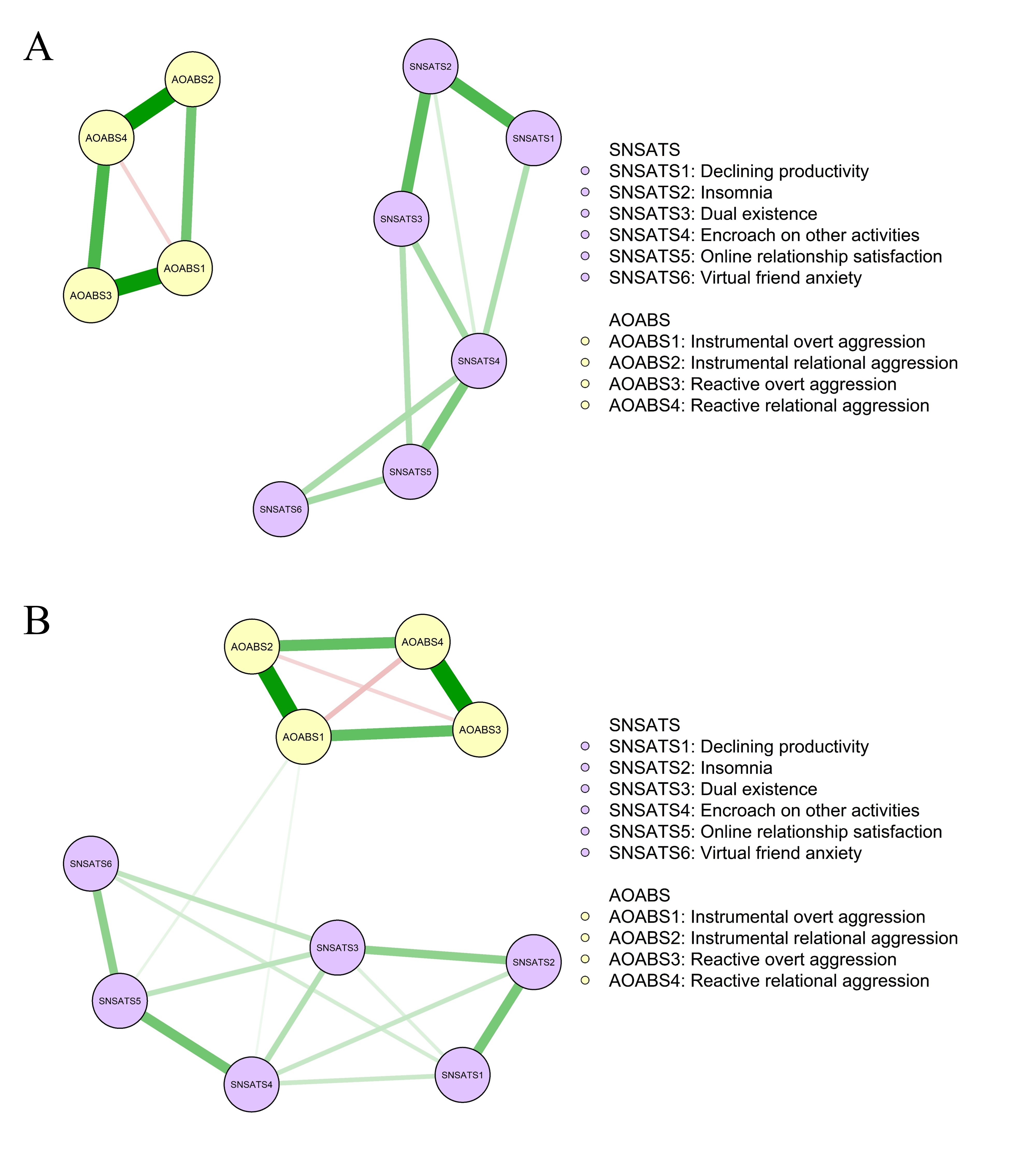


**Figure S1:** Network structure for males (*n* = 580; A) and females (*n* = 745; B) based the on the continuous data. Positive associations between symptoms are denoted by green lines, while negative associations are represented by red lines. Edge thickness indicates association strength. Mantel tests revealed a strong correlation between the edge-weight matrices of the dichotomized and continuous networks in both males (*r* = .86, *p* = .001) and females (*r* = .87, *p* = .001), indicating that dichotomized networks captured the underlying relationships similarly to the continuous ones.


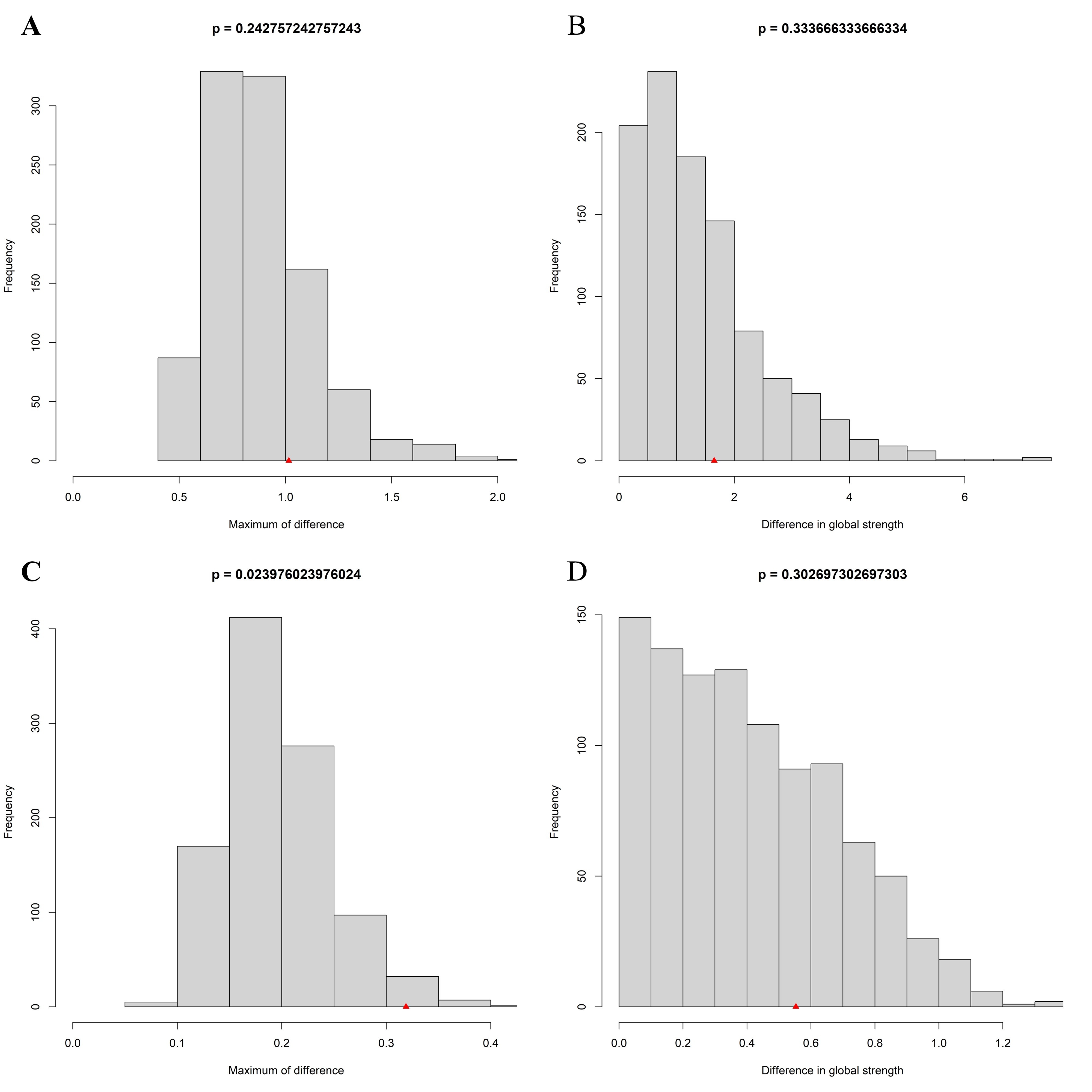


**Figure S2.** The gender differences in network structure and global strength. Panels A and B depict the Ising network differences based on the binary data, while panels C and D depict the network differences based on the continuous data.

**
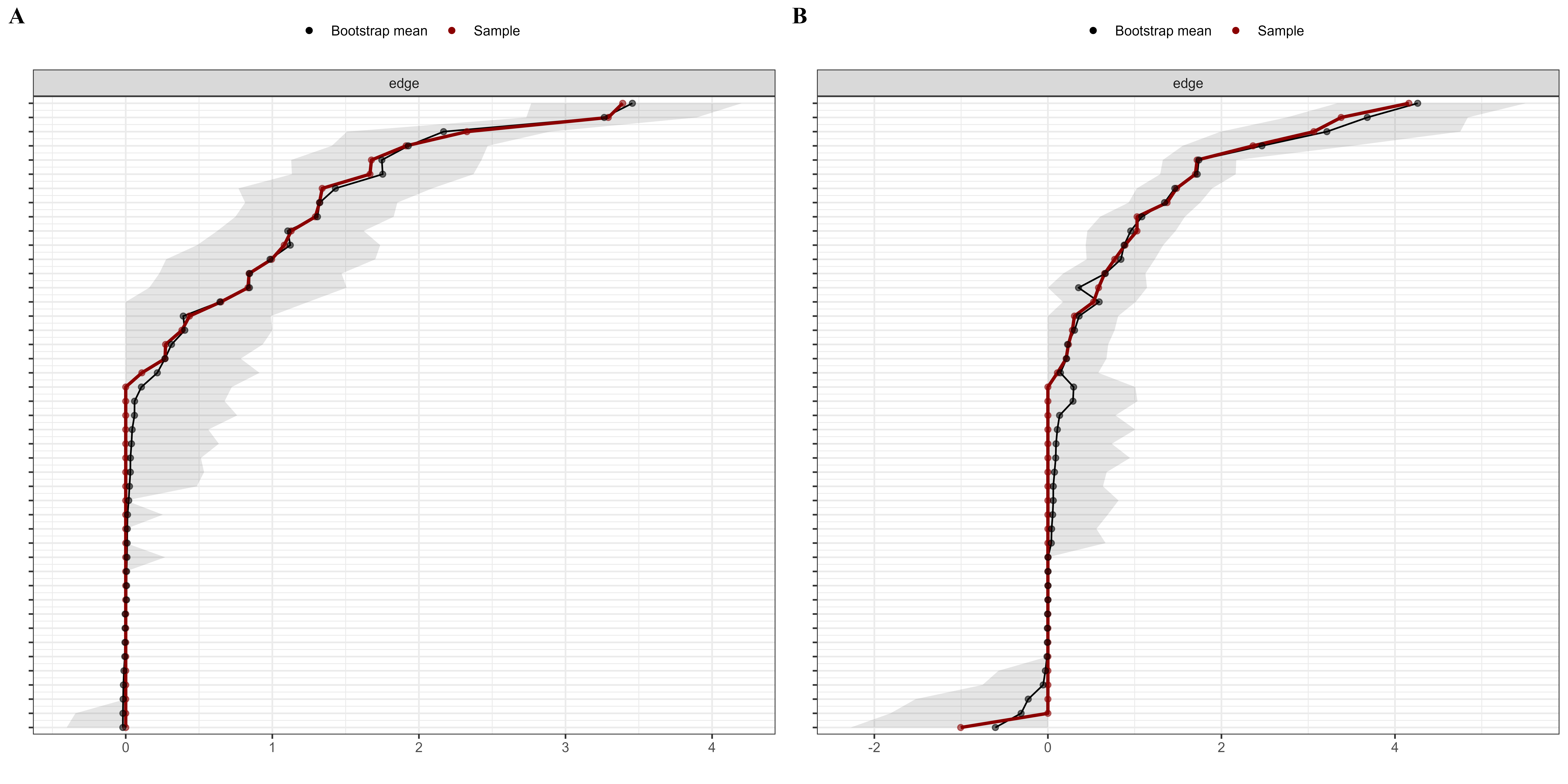
**

**Figure S3.** Nonparametric bootstrapped confidence intervals of estimated edges for males (A) and females (B). The red line represents the estimated edge, while the dark area indicates the 95% bootstrap confidence interval.


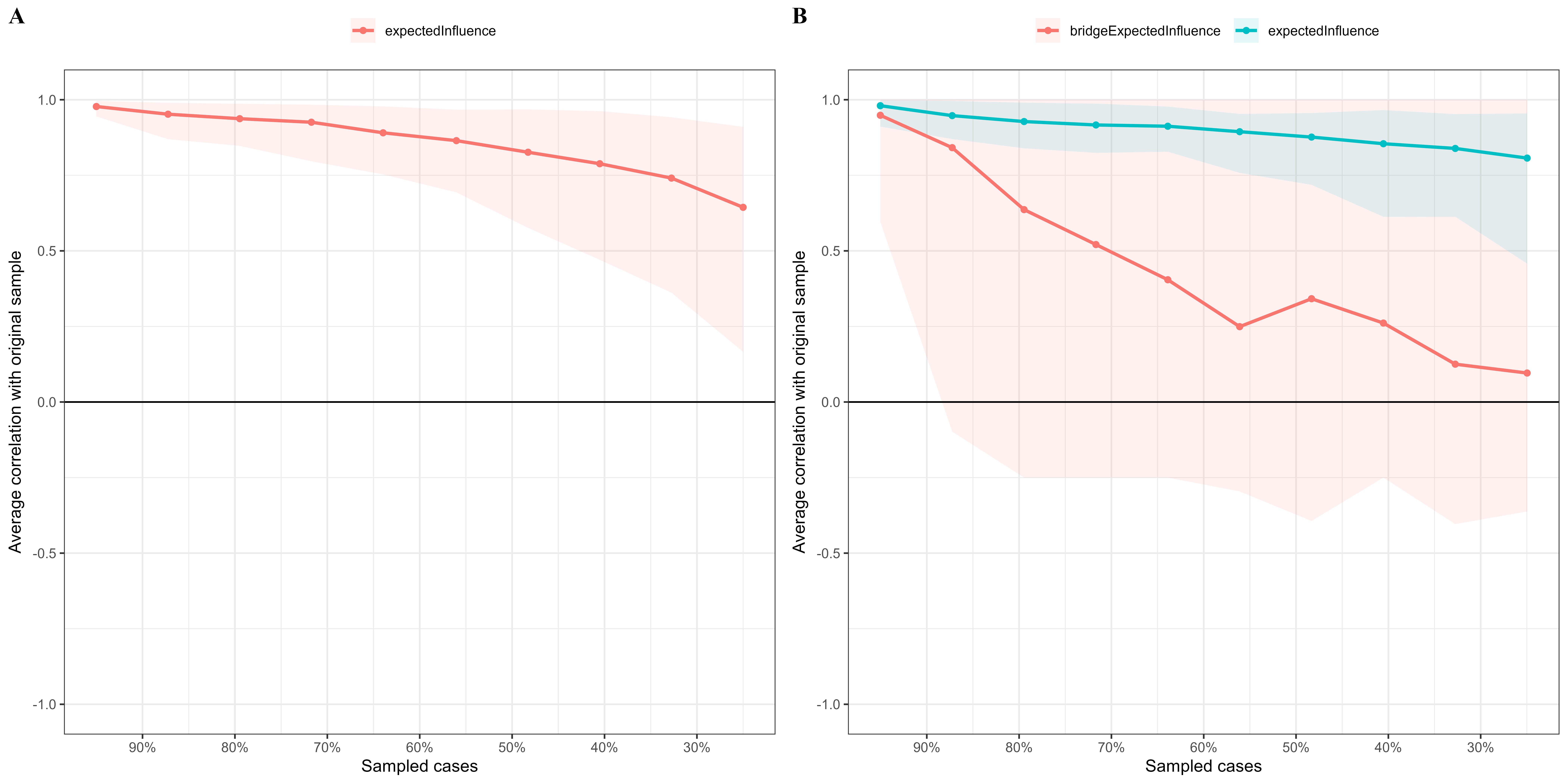


**Figure S4**. Case-dropping bootstrap test of centrality indices for males (A) and females (B). The x-axis indicates the percentage of cases of the original sample included at each step. The y-axis indicates the correlations between the centrality indices from the original network and the indices from the networks re-estimated after excluding increasing percentages of cases. In the estimation, nBoots = 1000, caseN = 40.

**
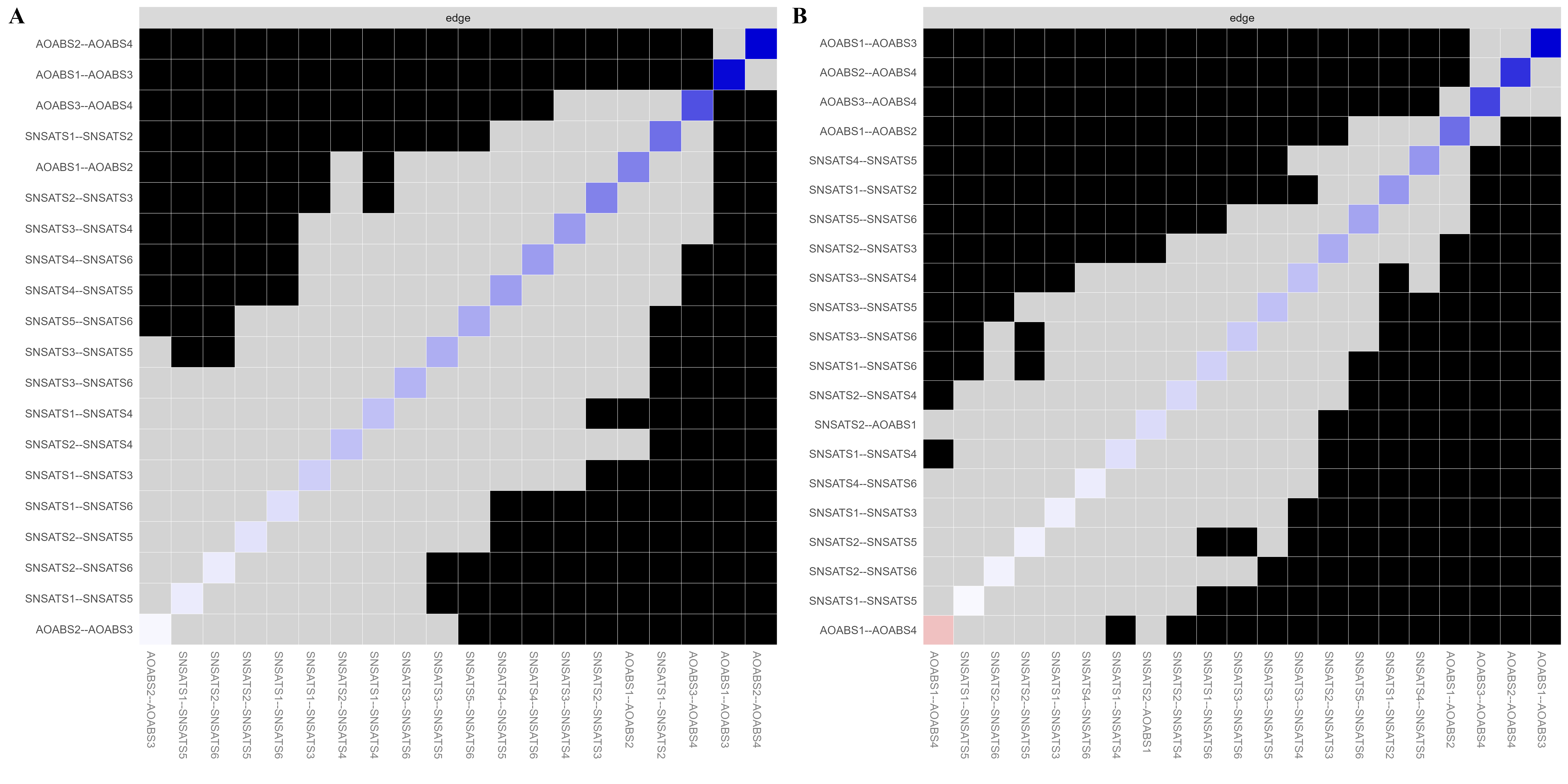
**

**Figure S5.** The results of the bootstrapped difference tests (*α* = 0.05) for pairwise edge weights for males (A) and females (B). The color of the boxes indicates whether edge-weights differ significantly from each other (i.e., black) or do not differ significantly (i.e., grey).

**
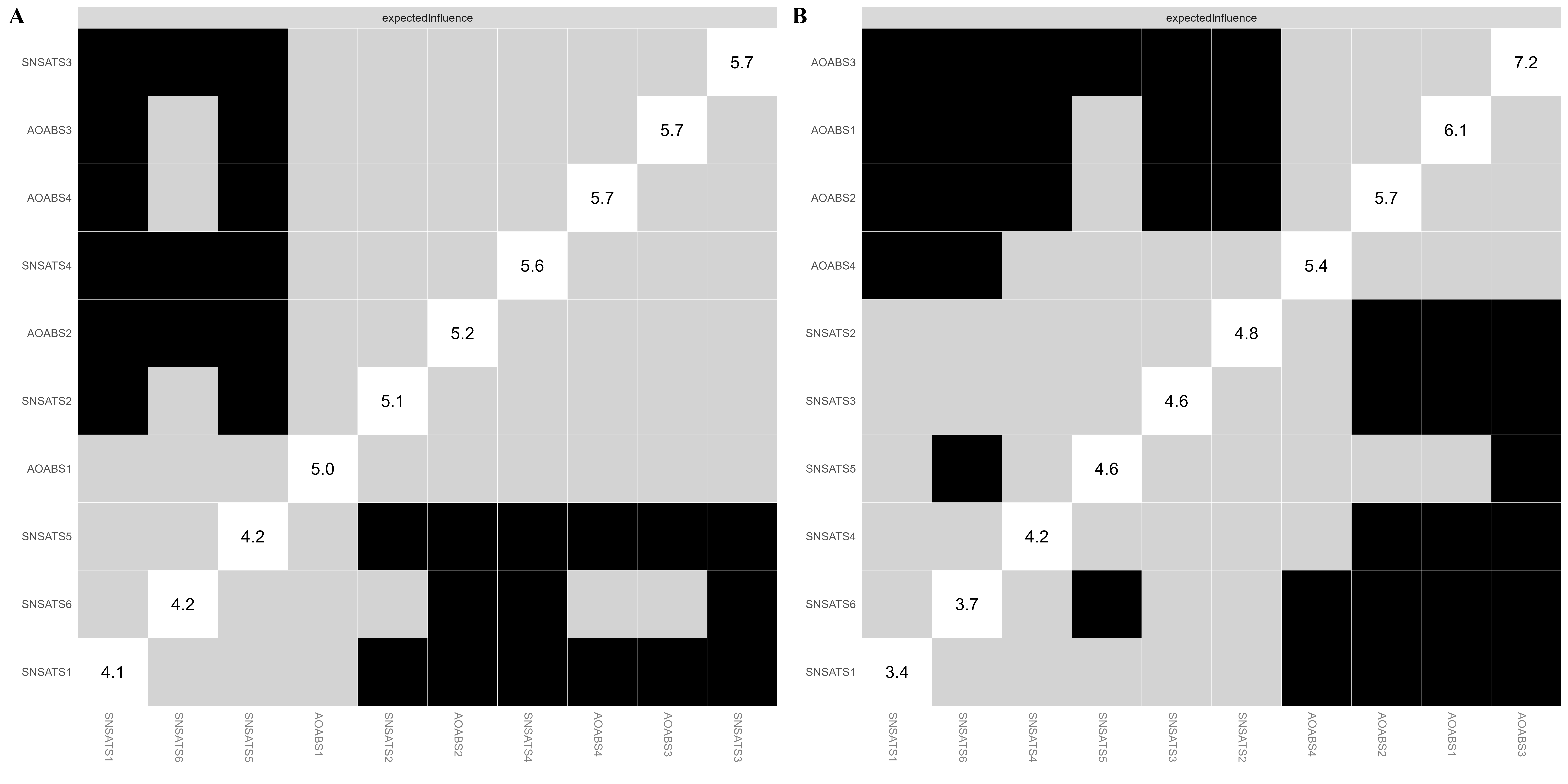
**

**Figure S6.** Nonparametric bootstrapped difference test for each node’s EI centrality indices in male (A) and female (B) networks. Grey boxes indicate no significant difference, whereas black boxes indicate a statistically significant difference (*p* < 0.05).

**
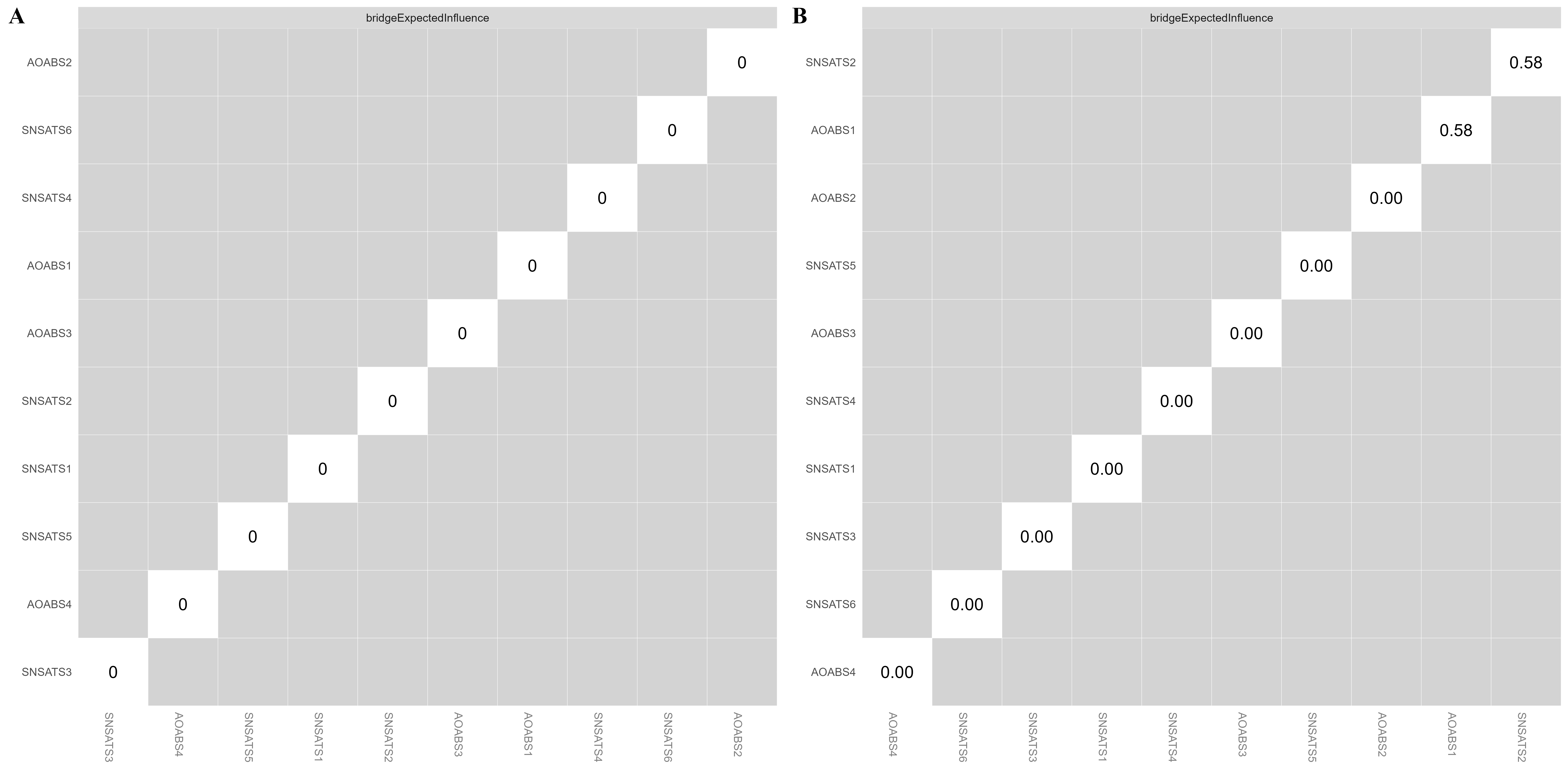
**

**Figure S7.** Nonparametric bootstrapped difference test for each node’s bridge EI centrality indices in male (A) and female (B) networks. Grey boxes indicate no significant difference, whereas black boxes indicate a statistically significant difference (*p* < 0.05).
